# The clinical diagnosis of pelvic inflammatory disease – reuse of electronic medical record data from 189 patients visiting a Swedish university hospital emergency department

**DOI:** 10.1186/1472-6874-6-16

**Published:** 2006-10-21

**Authors:** Jan Eggert, Kristina Sundquist, Caroline van Vuuren, Aino Fianu-Jonasson

**Affiliations:** 1 Department of Family and Community Medicine, Karolinska Institute, Huddinge, Stockholm, Sweden; 2 Department of Obstetrics and Gynecology, Karolinska University Hospital Huddinge and Karolinska Institute, Huddinge, Stockholm, Sweden

## Abstract

**Background:**

The pelvic inflammatory disease (PID) diagnosis is mostly based on clinical findings. However, few studies have examined the clinical basis for the diagnostics of PID, which was the aim of this study.

**Methods:**

A retrospective study was performed of 189 out-patients diagnosed as having PID at the obstetric and gynecological emergency department of a Swedish university hospital. Data on symptoms, signs, pelvic examination and laboratory tests were extracted from the electronic medical records in comparison with the diagnostic criteria of the PID Guideline of the US Center of Disease Control from 2002 (CDC 2002 Guidelines).

**Results:**

Eight symptoms in varying combinations were associated with the PID diagnosis. Most of them are mentioned in the CDC 2002 Guidelines. Detected rates of C. Trachomatis (CT) and N. Gonorrhoeae (NG) were 5% and 0%, respectively, among the tested patients (CT = 52% and NG = 12%). The C-reactive protein was normal in the majority of tested patients.

**Conclusion:**

The clinical basis for the diagnostics of PID was largely in accordance with the criteria in the CDC 2002 Guidelines. The limited number of CT tests performed is somewhat disappointing, considering the fact that effective disease prevention includes widespread CT screening. Further studies in different settings are needed in order to analyze how the testing rate for CT can be improved in clinical praxis.

## Background

Pelvic inflammatory disease (PID) includes endometritis, salpingitis, tubo-ovarian abscess, and/or pelvic peritonitis [1]. PID is a relatively common disease; in the U.S. around 8% of all women will have PID during their reproductive period [2], and in the U.K. PID contributes to about 2% of the yearly visits in general practice [3,4]. Causative agents are the sexually transmitted infections C. Trachomatis and N. Gonorrhoeae but may include several other bacteria, such as anaerobic Gram-negative rods and Mycoplasma Genitalium [5,6]. The complications of PID include infertility and ectopic pregnancy, which in turn causes substantial medical and social consequences for the affected individuals.

In addition, the financial burden on society for infertility examinations and treatments is considerable [7]. Diagnosis of PID is difficult because symptoms among women with PID vary widely from severe to none. For example, as many as 50% of women with tubal disease at infertility examination or ectopic pregnancy report having had no PID [8]. Moreover, some of these women have visited doctors for lower abdominal symptoms, not recognized as PID [9].

National Guidelines for PID from the US Center of Disease Control and Prevention (CDC 2002 Guidelines) gradually modified the criteria for clinical diagnosis in 1998 and 2002 in order to cover milder forms of PID [1,10]. The aim was to minimize missed diagnoses and reduce serious complications of PID. Clinicians are recommended to apply a low threshold for treatment of suspected cases. The current criteria proposed by CDC 2002 Guidelines imply maximal sensitivity, while partly sacrificing specificity. However, the low threshold for treatment of suspected cases should be maintained due to the severe complications of undetected PID, and because mild, atypical and silent cases of PID are common, expressed in the U.K. Prodigy guidance on PID as "Many women have no symptoms" [4].

The clinical diagnosis of acute PID is imprecise and has a positive predictive value for salpingitis of 65% to 90% compared with laparoscopy [1]. However, for most patients with PID, laparoscopic examination will not be applicable for practical and economic reasons. Therefore, the PID diagnosis will in the large majority of cases be based on clinical findings.

The aim of this retrospective study was to describe the clinical basis for the diagnostics of PID in a Swedish hospital setting and compare them with the diagnostic criteria of the CDC 2002 Guidelines. For this purpose we determined the frequency of reported symptoms and signs, and performed examinations among all out-patients who were clinically diagnosed with PID at the obstetric and gynecological emergency department (OGED) in a Swedish university hospital during 2001.

## Methods

All 189 out-patients with clinical PID were diagnosed in OGED at the Karolinska University Hospital, site Huddinge, which gives service to 350,000 inhabitants in southern Stockholm. Referral by another doctor is not needed. Almost all Swedish health care is financed by the taxpayers and the patient fee is low. Thus, the OGED receives a very high proportion of women with lower abdominal pain, bleedings, abnormal discharge, and acute genital infections.

The medical history data were collected by the gynecologist on duty. No uniform data collection form was used. A pelvic examination, i.e. a bimanual vaginal palpation, was performed in all cases followed by, in 82% of the cases, a transvaginal sonography (TVS). The time available was approximately 20–25 minutes.

There were 4,671 gynecological patients treated at the OGED in 2001 and we checked all their ICD-10 codes (International Classification of Diseases, tenth revision) in the electronic medical records (EMR). There were 381 patients with ICD-10 codes for PID (N70.0–N71.9; N73.0–N73.5). Women with an obvious cause of their PID were excluded, i.e. women with PID following partus, miscarriage, abortion, insertion of IUD and intra-uterine operations (n = 175). Sixteen in-patients were also excluded. The inclusion criteria gave 189 out-patients with PID diagnosis for the study.

We extracted from all 189 patient's EMR background data (age, parity, contraceptive method) length of history, symptoms and signs, pelvic examination, laboratory tests and medication. The length of history was determined by the symptom with the longest reported duration.

For each laboratory test, there were tested and non-tested patients. C-reactive protein (CRP) was analyzed via a capillary test. A urine specimen was requested from all patients before the pelvic examination and 52% were able to produce it (n = 98). The urine was analyzed for leucocyturia, measured by a test stick.

Urine culture was performed in 28 patients, probably when there was a suspicion of a combination of PID and UTI.

C. Trachomatis (CT) was analyzed with a polymerase chain reaction (PCR) method (Roche COBOS Amplicor^®^). A swab was taken always from the endocervix and mostly from the urethra. N. Gonorrhoeae (NG) was cultured via an endocervical swab.

### Ethics

This study was approved by the Ethics Committee at Karolinska Institutet, Stockholm, in 1999/2000.

## Results

More than half of the 189 patients were 30 years old or younger and 36% were childless. Twenty-four percent had an IUD. More than 50% of the patients had a length of history up to one week, and around 20% more than one month (Table 1). Thirty-nine patients had visited a doctor, mostly a GP, within days or weeks before they came to the OGED. Fifteen of those had been treated for suspected UTI and another 15 patients had visited a gynecologist, without receiving PID treatment (data not shown).

**Table 1:** Age, parity, contraceptive method, and length of history (in percentages) among the 189 patients with diagnosed PID.

	**Percentage**
**Age (years)**	
15–20	13
21–30	40
31–40	29
> 40	18
	
**Parity**	
0-para	36
1-para	15
2-3-para	36
≥4-para	4
Unknown	9
	
**Contraceptive method**	
IUD	24
Oral contraception	18
Sterilization or long-acting progesterone	8
No specified method	50
	
**Length of history**	
1–7 days	52
1–2 weeks	6
2–4 weeks	24
1–3 months	15
3–6 months	4
> 6 months	2

Abdominal pain was reported by 98%, abnormal vaginal discharge by 45%, while feelings of sickness, dysuria, fever and/or chills, lower back pain, and abnormal bleedings were each reported by 24–30% of the patients. Pain at intercourse was reported by 5%. All 189 patients were tender at examination. Almost half of the patients had tenderness over the uterus and/or motion tenderness of cervix and both adnexa, 20% were tender over the uterus and/or cervix only, 19% over the uterus and one of the adnexa and 12% over the adnexa only (Table 2).

**Table 2:** Frequency (percentages) of the symptoms and signs and tenderness at examination among the 189 patients diagnosed with PID.

	**percentage**
**Symptoms **	
Abdominal pain	98
Abnormal vaginal discharge	45
Feelings of sickness	30
Dysuria	27
Fever and/or chills	25
Lower back pain	25
Abnormal bleedings	24
Pain at intercourse	5
	
**Tenderness at examination**	
Uterus and/or motion of cervix and both adnexa	49
Uterus and/or motion of cervix only	20
Uterus and/or motion of cervix and one adnexa	19
Both adnexa (uterus untender)	6
One adnexa (uterus untender)	6

CT was detected among 5% of the 98 tested patients, while no NG was detected among the 23 tested patients. The CRP was normal (< 10) among more than 50% of the 110 tested patients. About 50% of the 98 patients who were able to produce a urine test had leucocyturia.

Urine culture was positive (> 100,000 bacteria per ml) among 6 of the 28 tested patients with suspicion of having both PID and UTI. TVS was performed in 156 patients. Ovarial cysts were found in 15% of them and in 5% there was pelvic fluid (Table 3).

**Table 3:** Results of the laboratory tests and the transvaginal sonography among the tested subgroups of the 189 patients diagnosed with PID.

	**percentage**
**C-reactive protein n = 110/189**	
< 10 (normal)	55
≥10(elevated)	45
	
**Urine leucocytes n = 98/189**	
Negative	54
Positive	46
	
**C. Trachomatis n = 98/189**	
Positive	5
	
**N. Gonorrhoeae n = 23/189**	
Positive	0
	
**Transvaginal sonography n = 156/189**	
Ovarial cysts	
no cysts	85
cyst < 10 mm	3
cyst < 20 mm	8
cyst < 21–40 mm	3
cyst > 41 mm	1
Fluid	
Yes	5
No	95

Wet smear was performed among less than 20% of the patients. All 189 patients were given oral treatment with antibiotics; 55% received doxycycline and metronidazol, 23% received cefuroxim or cefalexin and metronidazol, and 22% were given other antibiotic treatment (data not shown).

## Discussion

The main finding of this study was that the clinical basis for the diagnostics of PID was largely in accordance with the symptoms and signs mentioned in the CDC Guidelines. For example, clinical symptoms mentioned in the CDC 2002 Guidelines as criteria for diagnosis are abdominal pain, abnormal vaginal discharge and fever, while abnormal bleedings and pain at intercourse are mentioned as possible, albeit non-specific PID symptoms.

The above-mentioned symptoms from the CDC 2002 Guidelines were all reported among the patients in our study. The most frequent symptom was abdominal pain, followed by abnormal vaginal discharge. Other common symptoms were fever and/or chills and abnormal bleedings, whereas pain at intercourse was a rarer symptom. In addition, feelings of sickness, dysuria, and lower back pain were all frequent symptoms among our patients, although not mentioned in the CDC 2002 Guidelines.

CDC 2002 Guidelines recommend antibiotic treatment of suspected PID if (1) cervical motion tenderness or (2) uterine or adnexal tenderness is present and no other cause is identified. The doctors in this study performed a bimanual examination on all 189 patients, and found that all had cervical motion tenderness or were tender over the uterus and/or the adnexa. In addition, 19% of the patients were tender over the uterus and the adnexa, and 20% had only uterine tenderness, which is in agreement with the CDC 2002 Guidelines and with some previous studies [11,12].

CDC 2002 Guidelines do not mention the degree of tenderness, which is understandable considering its subjective dimension. However, most of the doctors that examined the patients in this study used three degrees of assessed tenderness in the bimanual examination; light, moderate and pronounced. A few previous PID studies have used a severity score of tenderness with three degrees in treatment evaluations, including the recent large-scale PEACH study [13-15].

Additional diagnostic criteria in the CDC guidelines include the presence of white blood cells on wet smear, elevated erythrocyte sedimentation rate, elevated C-reactive protein, a positive culture of CT or NG and a TVS that shows fluid-filled thickened tubes, with or without pelvic fluid.

In our study wet smear was performed among less than 20% of the patients. Erythrocyte sedimentation rate was not used in the diagnostic procedure, possibly because it has largely been replaced by C-reactive protein in acute medical consultations in Sweden. Most of the 110 tested patients had normal or low CRP. In the CDC 2002 Guidelines an elevated CRP is an additional criterion supporting a PID diagnosis, while a normal CRP cannot exclude it [16].

About half of the patients were tested for CT, giving a prevalence rate among the tested patients of 5%. The low testing rate for CT observed in the present study is somewhat disappointing considering the fact that summarized studies of the cost effectiveness of screening for CT have shown that it is cost-saving to overtest symptomatic women at a prevalence as low as 1.1% [17]. In addition, a study from 1996 found evidence that screening for CT had reduced PID in women by as much as 60% [18]. The low testing rate for CT in the present study could have resulted in inadequate treatment of partners and the subsequent reinfection of some women after antibiotic treatment.

However, the relatively low detected rate of CT (5%) among the tested patients is in accordance with other Swedish studies of the prevalence of CT. In a Swedish longitudinal study, CT rates in PID patients in a hospital setting decreased from 17% in 1988 to < 5% in 1997 [19,20].

It has been mandatory by law since 1988 to report positive CT cases to the Stockholm Regional Department of Communicable Diseases, Control and Prevention [21]. However, despite (or perhaps even because of) mandatory notification of CT cases, doctors did not test a sufficient proportion of the PID patients for CT.

The low testing rate for NG follows Swedish traditions, i.e. testing mainly patients with pronounced risk behavior, at the patient's own request, when tracing contacts and after therapy failure in patients with PID and urethritis. In a Swedish study, the rates of NG in PID patients treated in hospital care was 0% in both 1988 and 1997 [19,20].

In contrast, a study from the U.K. found that the rates of gonococcal PID were increasing [22]. A systematic review from the UK found that healthcare settings had higher CT rates than population-based studies, where the overall prevalence was 5% [23]. However, in some settings in the U.S. and in the UK, the STI rates are considerably higher. Studies of PID patients have shown that the CT and NG rates are 15–30% and 10–20%, respectively; however, this is mainly in inner-city settings [15,22]. In Antwerp, Belgium, the overall prevalence of CT among sexually active women was 5% [24], which is in accordance with the present study.

The prevalence of CT varies with age, i.e. younger women have a higher prevalence rate of CT than older women [23]. We examined possible differences between women that were tested for CT and women that were non-tested. Tested women had a mean age of 27 years and non-tested of 35. Older women were possibly considered less important to test.

TVS was performed among a large proportion of our patients, possibly to exclude differential diagnoses. It is possible that TVS was overused, because TVS cannot rule out mild to moderate PID, and none of our patients had TVS-specific signs [25] at the TVS examination. A study from Finland found that Power Doppler TVS was 100% sensitive and 80% specific in the diagnosis of PID, i.e. the overall accuracy was 93% [25]. Other studies have confirmed the usefulness of TVS in the diagnosis of PID [26-29]. However, usefulness is probably highly related to each doctor's experience with using TVS in the diagnosis of PID.

Dysuria is not mentioned as a diagnostic criterion of PID in the CDC 2002 Guidelines. However, it is mentioned as a PID criteria in the CDC Pelvic Inflammatory Disease – CDC Fact Sheet for the public 2004 [30]. In some of our patients dysuria may have been caused by e.g. undiagnosed UTI or the PID itself.

Time aspects are not discussed in the CDC 2002 Guidelines. In this study, the majority had a short symptom history, which is in agreement with the PEACH study [15]. However, as many as 20% had a symptom history of more than one month and 21% had visited a doctor shortly before they came to the OGED, which suggests that some of these patients might, at least initially, have presented mild or atypical symptoms of PID.

The key strength of this study was that the retrospective design meant that the doctors' management of the patients was unbiased by, for example, any intervention. In addition, the sample size and the inclusion of relatively non-selected open-care patients could be seen as additional strengths of this study.

As inherent in all studies of clinically diagnosed PID, some of the patients in this study did not have PID. Additionally, our data did not allow us to include missed cases of PID, as the extracted electronic medical records of the 189 patients only included diagnosed cases of PID that were based on the ICD codes. However, the aim of this study was to describe the clinical basis for diagnosis and treatment among the 189 patients, and not to determine how many of them actually had PID. It is possible that the emergency ward environment may have affected the quality of the case notes, and that not all information that the doctors used for their management of the patients was included in the records.

The authors of a retrospective chart review set out to determine whether emergency department practitioners at an urban teaching hospital in the US complied with CDC guidelines for diagnosing and treating sexually transmitted diseases. They found a number of deficits in the adherence to recommended guidelines [31]. A national study from England and Wales assessed the quality of the diagnosis and treatment of PID in general practice. The findings of that study reflected a low disease awareness and sub-optimal management of PID, which the authors concluded was a fundamental obstacle to effective disease intervention [32].

## Conclusion

The clinical basis for the diagnostics of PID was largely in accordance with the criteria in the CDC 2002 Guidelines. The limited number of CT tests performed (52%) is somewhat disappointing considering the fact that effective disease prevention includes widespread screening. In addition, some of our patients had a relatively long symptom history, which suggests that they might initially have presented mild or atypical symptoms of PID. Further studies could therefore focus on symptoms and signs in milder forms of PID, and how the testing rate for CT can be improved in clinical praxis.

## Competing interests

The author(s) declare that they have no competing interests.

## Authors' contributions

JE participated in the design of the study, acquisition of data, analysis and interpretation of data, drafting of the manuscript, critical revision of the manuscript, and supervision.

KS participated in the analysis and interpretation of data, drafting of the manuscript, critical revision of the manuscript, obtaining funding, supervision.

CVV participated in the acquisition of data, analysis and interpretation of data, drafting of the manuscript, and technical support.

AFJ participated in the design of the study, acquisition of data, analysis and interpretation of data, critical revision of the manuscript, and supervision.

All authors read and approved the final manuscript.

## Pre-publication history

The pre-publication history for this paper can be accessed here:


